# Burden of bacterial resistance among neonatal infections in low income countries: how convincing is the epidemiological evidence?

**DOI:** 10.1186/s12879-015-0843-x

**Published:** 2015-03-15

**Authors:** Bich-Tram Huynh, Michael Padget, Benoit Garin, Perlinot Herindrainy, Elsa Kermorvant-Duchemin, Laurence Watier, Didier Guillemot, Elisabeth Delarocque-Astagneau

**Affiliations:** Pharmacoepidemiology and Infectious diseases Unit, Institut Pasteur, UVSQ, EA 4499, Versailles, INSERM Unit 657, 25,28 rue du Docteur Roux, 75724 Paris, France; Experimental Bacteriology Laboratory, Institut Pasteur, Antananarivo, Madagascar; Epidemiology Unit Institut Pasteur, Antananarivo, Madagascar; Necker Hospital, Department of Neonatal medicine, Paris Descartes University, Paris, France; Pharmacoepidemiology and Infectious diseases Unit, Institut Pasteur, UVSQ, EA 4499, Versailles, INSERM Unit 657 Paris, France AP-HP, Hospital Raymond-Poincaré, Garches, France

**Keywords:** Antibiotic resistant bacterial infection, Developing countries, Neonatal, Epidemiology, Community

## Abstract

**Background:**

Antibiotic resistance is a threat in developing countries (DCs) because of the high burden of bacterial disease and the presence of risk factors for its emergence and spread. This threat is of particular concern for neonates in DCs where over one-third of neonatal deaths may be attributable to severe infections and factors such as malnutrition and HIV infection may increase the risk of death. Additional, undocumented deaths due to severe infection may also occur due to the high frequency of at-home births in DCs.

**Methods:**

We conducted a systematic review of studies published after 2000 on community-acquired invasive bacterial infections and antibiotic resistance among neonates in DCs. Twenty-one articles met all inclusion criteria and were included in the final analysis.

**Results:**

Ninety percent of studies recruited participants at large or university hospitals. The majority of studies were conducted in Sub-Saharan Africa (n = 10) and the Indian subcontinent (n = 8). Neonatal infection incidence ranged from 2.9 (95% CI 1.9–4.2) to 24 (95% CI 21.8–25.7) for 1000 live births. The three most common bacterial isolates in neonatal sepsis were *Staphylococcus aureus, Escherichia coli,* and *Klebsiella*. Information on antibiotic resistance was sparse and often relied on few isolates. The majority of resistance studies were conducted prior to 2008. No conclusions could be drawn on *Enterobacteriaceae* resistance to third generation cephalosporins or methicillin resistance among *Staphylococcus aureus*.

**Conclusions:**

Available data were found insufficient to draw a true, recent, and accurate picture of antibiotic resistance in DCs among severe bacterial infection in neonates, particularly at the community level. Existing neonatal sepsis treatment guidelines may no longer be appropriate, and these data are needed as the basis for updated guidelines. Reliable microbiological and epidemiological data at the community level are needed in DCs to combat the global challenge of antibiotic resistance especially among neonates among whom the burden is greatest.

## Background

Infectious disease remains the leading cause of death in children under 5 in developing countries (DCs) with neonates bearing the highest burden. In Africa alone, infectious disease accounts for over 76% of under-5 deaths, and an estimated 36% of neonatal deaths worldwide are directly attributable to severe infections [1,2].

Moreover, DCs are also home to a number of risk factors for the emergence and spread of antibiotic resistance. Misuse of antibiotics, over-the-counter and parallel market access, and counterfeit or poor quality drugs, combined with substandard hygiene and living conditions, are the driving forces behind the emergence and spread of antibiotic resistance [3,4]. The potential for the development and rapid spread of new forms of resistance is highlighted by the recent worldwide proliferation of NDM-1-producing *Enterobacteriaceae.* The gene, which confers resistance to carbapenems, originated in India in 2009, and since 2010 NDM-1-producing *Enterobacteriaceae* have been reported in North America, Europe, and Asia [5]. The World Health Organization (WHO) has recently heightened awareness of this pressing issue with calls for action to contain antibiotic resistance on a global scale [6].

A recent report estimates 6.9 million cases of possible severe bacterial infection in neonates in sub-Saharan Africa, south Asia, and Latin America in 2012 [7] underscoring the potential for excess morbidity and mortality due to antibiotic resistance. In this context, antibiotic resistance trends need to be monitored.

We report a systematic review examining studies dealing with invasive bacterial infections and antibiotic resistance among neonates in DCs with a special emphasis placed on community-based studies.

## Methods

An initial search was conducted for articles dealing with infection and antibiotic resistance among children under 2. This search allowed us to identify both neonatal-specific articles as well as articles dealing with young childhood infection that included neonatal-specific information.

We searched PubMed for studies published in 2000 or later (last search April 30th, 2014). To overcome a potential lack of studies dealing with both topics simultaneously, our search was divided into two branches including 1) community-acquired bacterial infections in infants of DCs (BI), and 2) antibiotic resistance among community-acquired infections in DCs (AR). DCs were identified as “least developed”, “other low income”, or “lower middle income” by the World Bank or as “low human development” or “medium human development” by the United Nations [8,9]. We also screened reference lists of relevant articles for further publications.

Before paper selection, duplicates from the BI and AR lists were eliminated. Detailed PubMed search and inclusion criteria are shown Table 1. Abstracts were screened for full text reading by two of the three reviewers (B-T.H., M.P., and E.D.A.) and a third reviewer was called upon as needed. Information was extracted from selected articles including: study year, study location, urban vs. rural location, hospital recruitment vs. other, community or nosocomial infections, study design and microbiology methods (bacterial isolation methods and antibiotic susceptibility testing). Articles were then re-evaluated by two reviewers as before.

**Table 1:** **Search strategy and selection criteria for neonatal infection and bacterial resistance articles in developing countries (2000-May 2014)**

**Search strategy**	
For the BI search, each DC was cross-linked with search terms “Bacterial Infections” OR “Sepsis” OR “bacter*” AND “epidemiology”. For the AR search, each DC was cross-linked with “Drug resistance, bacterial” OR (“antibiotic resistance” AND “bacter*”) AND “epidemiology”. Both searches were restricted to English language articles and the BI search was restricted to the PubMed “infant” age category (birth-23 months). Both searches were also limited by excluding the keywords and MeSH terms “travel”, “candida”, “HIV infection”, “leprosy”, “tuberculosis”, “tetanus”, “malaria”, “cholera”, or “helicobacter”. The BI search was further limited by excluding the keywords “immunization”, “immunization program”, and “vaccination”.	
**Inclusion criteria**	
***Infant infection search*** **(BI)**	***Resistance search*** **(AR)**
● Information on bacterial infections including either etiology or disease burden/incidence	● Bacterial pathogens
● Community acquired infections	● Community acquired infections
● Methodologically sound including clear inclusion criteria	● Information on antibiotic resistance profile of pathogen (proportion resistance/susceptible, etc.)
● Sound microbiological methods/citation of guidelines used	● Sound microbiological methods/citation of guidelines used
● Neonatal specific information presented	● Information on pathogen source and/or clinical information
	● Neonatal specific information presented
**Exclusion criteria**	
***Both branches***	
● Review study or expert opinion	
● Outside of developing country list	
● Purely nosocomial infections or no possibility to extract only community acquired infections from data	
● Pathogen not in the restricted list, including *Neisseria gonorrhoeae*, *Campylobacter*, *Helicobacter*, V*ibrio*, *Clostridium tetani*, or any Mycobacteria	
● Obvious methodological weakness including sampling methods	
● Insufficient number of isolates/insufficient number of isolates for follow-up period (minimum 10 isolates per year)	
● Data collection done principally before 2000	
***Infant infection search*** **(BI)**	***Resistance search*** **(AR)**
● Ages outside of range of interest or ages of interest non-extractable	● Insufficient epidemiological info on sample source/patients/no. of bacteria isolated from neonates

Following the selection of articles for children <2, those articles containing information on either bacterial infection or resistance during the neonatal period were retained for analysis. An effort was made to restrict the selection to community-acquired infections, and all articles presenting exclusively nosocomial infections were eliminated.

## Results

**Figure 1:**
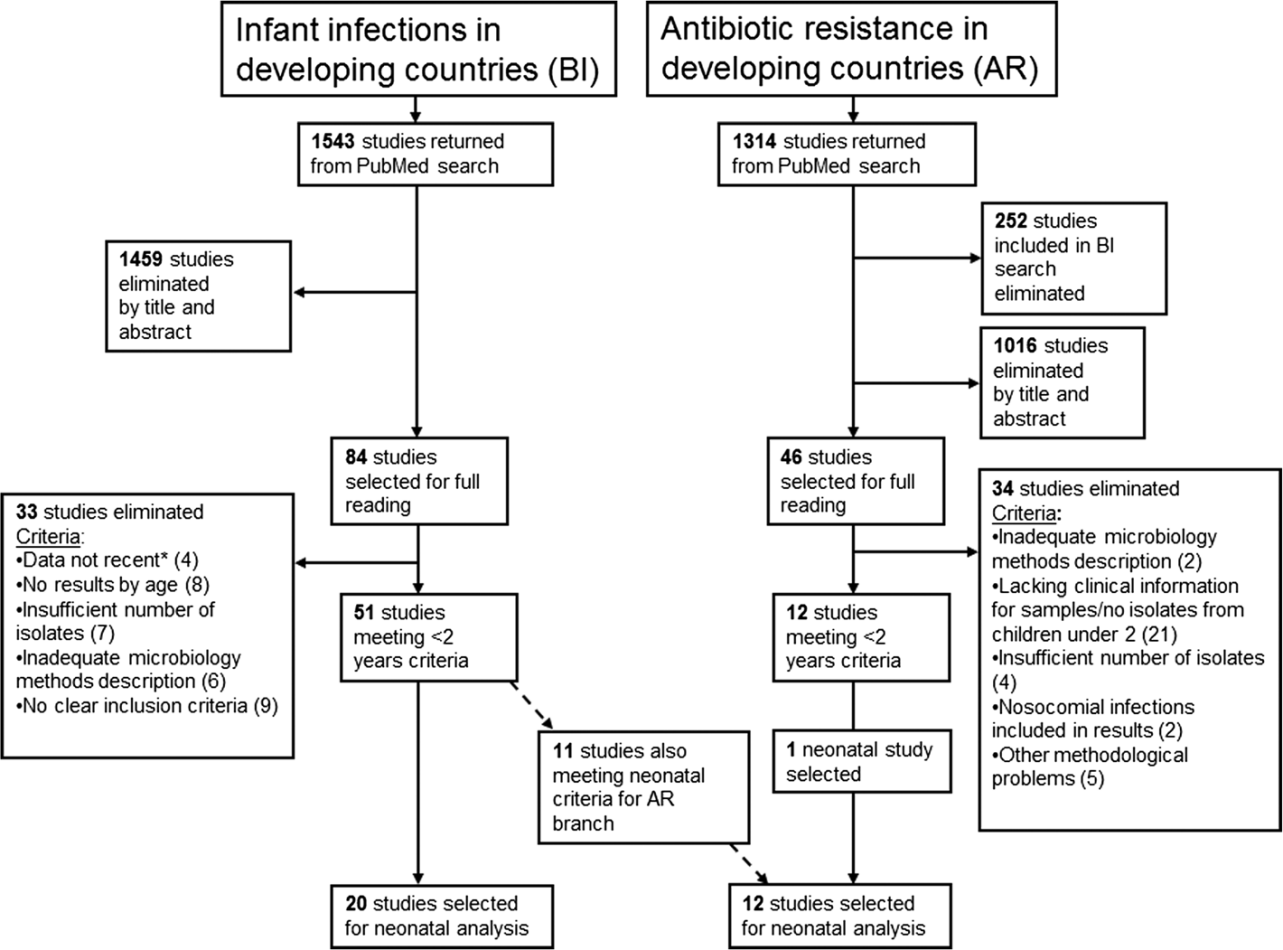
**Flowchart of literature search including both the infection incidence and antibiotic resistance branches.** *Data was considered not recent if data collection took place principally before 2000.

Of the 1543 and 1314 studies returned from the BI and AR searches, 84 and 46 were selected for full reading, respectively (Figure 1). Ultimately, 20 BI studies and one AR study were retained for a total of 21 studies included in the final analysis. Of the 20 BI studies selected, 11 also met the selection criteria for inclusion in AR studies and thus a total of 12 articles were included in the resistance analyses.

A majority of studies came from Sub-Saharan Africa (n = 10) or the Indian subcontinent (n = 8) (Table 2).

**Table 2:** **Neonatal infections in developing countries (2000-May 2014)**

**Author, country, and study year**	**Disease type and age**	**Setting**	Neonatal Isolation rate and aetiology*	
**Sub-Saharan Africa**				
Blomberg et al. [10]	*Bacteremia*	urban, hospital recruitment	**54 early onset (EOS) isolates** ^†^ **: 31 late onset (LOS) isolates:**	
Tanzania 2001-2002	<7 yrs		*Klebsiella* spp.	EOS 14 (26%), LOS 7 (23%)
			*S. aureus*	EOS 6 (11%), LOS 5 (16%)
			*E. coli*	EOS 6 (11%), LOS 3 (10%)
			Group B *Streptococcus*	EOS 2 (4%), LOS 1 (3%)
Sigaúque et al. [21]	*Bacteremia*	rural, hospital recruitment	**154 isolates: 16% blood cultures positive**	
Mozambique 2001-2006	<15 yrs		*S. aureus*	60 (39%)
			Group B *Streptococcus*	31 (20%)
			*E. coli*	9 (6%)
			*S. pneumoniae*	7 (5%)
Nielsen et al. [17]	*Bacteremia*	rural, hospital recruitment	**23 isolates:**	
Ghana 2007-2009	<5 yrs		*S. aureus*	6 (26%)
			*Klebsiella* spp.	6 (26%)
			*Streptococcus* spp.	3 (13%)
			*E. coli*	3 (13%)
			Non-tyhoid *Salmonella*	2 (9%)
Gray et al. [29]	*Group B streptococcus*	urban, hospital recruitment	**290 isolates: 12% blood cultures positive**	
Malawi 2004-2005	<90 days		Group B *Streptococcus*	48 (17%)
Talbert et al. [24]	*Neonatal sepsis*	rural, hospital recruitment	**474 isolates: 9% blood cultures positive (**25 infants had 2 bacterial species isolated)	
Kenya 2001-2009	<60 days		*Klebsiella* spp.	57 (13%)
			*S. aureus*	55 (12%)
			*Acinetobacter* spp.	48 (11%)
			*E. coli*	41 (9%)
			Group B *Streptococcus*	32 (7%)
			**86 isolates from CSF samples : 4% CSF cultures positive**	
			*S. pneumoniae*	17 (20%)
			Group B *Streptococcus*	16 (19%)
			*Salmonella* spp.	10 (12%)
Ojukwu et al. [18]	*Neonatal sepsis*	urban, hospital recruitment	**33 isolates: 24% blood cultures positive**	
Nigeria 2002-2003	0-28 days		*S. aureus*	15 (45%)
			*E. coli*	6 (18%)
			*Klebsiella* spp.	3 (9%)
			Group B *Streptococcus*	1 (3%)
Mugalu et al. [15]	*Neonatal sepsis*	urban, hospital recruitment	**110 isolates: 37% blood or CSF cultures positive**	
Uganda 2002	used WHO guidelines		*S. aureus*	69 (63%)
			*E. coli*	17 (15%)
			Group B *Streptococcus*	7 (6%)
Shitaye et al. [19]	*Neonatal sepsis*	urban, hospital recruitment	**135 isolates: 45% blood cultures positive**	
Ethiopia 2006-2007	0-28 days		*Klebsiella* spp.	53 (39%)
			*S. aureus*	30 (22%)
			Coagulase-negative *Staphylococcus*	10 (7%)
Mhada et al.	*Neonatal sepsis*	urban, hospital recruitment	**52 early onset (EOS) isolates** ^**†**^ **: 22 late onset (LOS) isolates: 22.4% blood cultures positive**	
Tanzania 2009-2010	*0-28 days*		*S. aureus*	EOS 15 (29%), LOS 12 (55%)
			*Klebsiella* spp.	EOS 17 (33%), LOS 5 (23%)
			*E. coli*	EOS 10 (19%), LOS 4 (18%)
			*Staphylococcus* epidermidis	EOS 6 (12%), LOS 0 (0%)
			Group B *Streptococcus*	EOS 1 (2%), LOS 0 (0%)
Kiwanuka et al. [13]	*Neonatal sepsis*	urban, hospital recruitment	**19 early onset (EOS) isolates** ^**†**^ **: 7 late onset (LOS) isolates: 33% blood cultures**	
Uganda 2010	<1 month		*S. aureus*	EOS 13 (68%), LOS 3 (43%)
			*E. coli*	EOS 3 (16%), LOS 1 (14%)
			*Klebsiella* spp.	EOS 1 (5%), LOS 1 (14%)
			Group B *Streptococcus*	EOS 1 (5%), LOS 0 (0%)
**SE Asia**				
Stoesser et al. [22]	*Bacteremia*	urban, hospital recruitment	**65 isolates:**	
Cambodia 2007-2011	<16 yrs		*Klebsiella* spp.	14 (22%)
			*S. aureus*	9 (14%)
			*Enterobacter* spp.	4 (6%)
			*E. coli*	3 (5%)
			*Streptococcus* pyogenes	3 (5%)
Kruse et al. [30]	*Neonatal sepsis*	urban, hospital recruitment	**399 isolates: 17% blood cultures positive**	
Vietnam 2009-2010	<28 days		*Klebsiella* spp.	78 (20%)
			*Acinetobacter* spp.	58 (15%)
			*E. coli*	21 (5%)
			*Enterobacter* spp.	16 (4%)
			*S. aureus*	11 (3%)
			*Morganella* spp.	8 (2%)
			*Pseudomonas* spp.	6 (2%)
			Coagulase-negative Staphylococcus	175 (44%)
**India subcontinent**				
Mir et al. [28]	*Omphalitis with sepsis*	urban, community recruitment	**432 isolates: 64% umbilical cord cultures positive**	
Pakistan 2004-2007	neonates (<1 month)		*S. aureus*	225 (52%)^‡^
			*Streptococcus* pyogenes	78 (18%)^‡^
			Group B *Streptococcus*	43 (10%)^‡^
Jain et al. [26]	*Neonatal sepsis*	urban, hospital recruitment	**350 isolates: 48% blood cultures positive for bacteria**	
India 2001-2002	Not defined		*Klebsiella* spp.	86 (25%)^‡^
			*Enterobacter* spp.	80 (23%)^‡^
			*E. coli*	49 (14%)^‡^
Sundaram et al. [23]	*Neonatal sepsis*	urban, hospital recruitment	**527 early onset (EOS) isolates** ^**§**^ **: 364 late onset (LOS) isolates:**	
India 1995–1998, 2001-2006	Not defined		*S. aureus*	EOS 108 (20%), LOS 112 (31%)
			*K. pneumoniae*	EOS 62 (12%), LOS 49 (14%)
			Non-fermenting gram negative bacilli	EOS 161 (30%), LOS 60 (17%)
			*E. coli*	EOS 48 (9%), LOS 40 (11%)
Zakariya et al. [25]	*Neonatal sepsis*	urban, hospital recruitment	**50 isolates: 42% blood cultures positive**	
India 2004-2006	<= 30 days		*K. pneumoniae*	33 (66%)
			Coagulase-negative Staphylococcus	6 (12%)
			Group B *Streptococcus*	1 (2%)
Muhammad et al. [16]	*Neonatal sepsis*	urban, hospital recruitment	**130 isolates:**	
Pakistan 2009-2010	<28 days		*S. aureus*	35 (27%)
			*E. coli*	30 (23%)
			*Staphylococcus* epidermidis	17 (13%)
			*Acinetobacter* spp.	17 (13%)
			*Klebsiella* spp.	13 (10%)
			*Streptococcus* species only found in early onset sepsis (first week)	
			*Klebseilla* species only found in late onset sepsis (after first week to 28 days)	
Darmstadt et al. [27]	*Neonatal sepsis*	rural, community recruitment	**29 isolates: 6% blood cultures positive**	
Bangladesh 2004-2006	<28 days		*S. aureus*	10 (34%)
			*S. pneumoniae*	3 (10%)
			Group B *Streptococcus*	1 (3%)
Gyawali et al. [12]	*Neonatal sepsis*	urban, hospital recruitment	**238 isolates: 15% blood cultures positive**	
Nepal 2009-2010	first 4 weeks of life		*S. aureus*	94 (40%)
			*Klebsiella* spp.	32 (14%)
			*Acinetobacter* spp.	30 (13%)
			*Enterobacter* spp.	27 (11%)
			*Pseudomonas* spp.	21 (9%)
			*E. coli*	16 (7%)
Shresta et al. [20]	*Neonatal sepsis*	urban, hospital recruitment	**37 isolates: 32% blood cultures positive**	
Nepal, 2011-2012	not defined		*S. aureus*	21 (57%)
			*K. pneumoniae*	8 (22%)
			*P. aeruginosa*	5 (13%)
**Europe**				
Macharashvili et al. [14]	*Neonatal sepsis*	urban, hospital recruitment	**126 isolates: 67% blood cultures positive**	
Georgia 2003-2004	8 weeks or younger		*K. pneumoniae*	36 (29%)
			*Enterobacter cloacae*	19 (15%)
			*S. aureus*	15 (12%)
			Group B *Streptococcus*	6 (5%)

*Percentages calculated when not reported in the article. Pathogens listed in order of relative percentages.

^†^Early onset sepsis (EOS) defined as 0–6 days.

^‡^Number of isolates calculated from percentages presented in article.

^§^Early onset sepsis (EOS) defined as <72 hours, late onset (LOS) defined as >72 hours.

### Design, recruitment settings, and study topics

Of the 21 articles analyzed, 17 used either a cross-sectional or surveillance study design [10-26]. Four studies were conducted in rural settings [17,21,24,27] while the remaining 17 were conducted in urban settings (Table 2).

Nineteen of 21 studies recruited participants in large district or university hospitals. Only two studies used active community recruitment [27,28]. Sixteen of the 17 urban studies recruited uniquely at large hospitals.

### Bacterial isolation rates

Thirteen of the 20 infant infection articles reported bacteremia rates. Excluding one study from Georgia with an isolation rate of 67% [14], isolation rates ranged from 5.8% to 48% (median = 22.4%). No difference in rates was noted between urban and rural studies [11-15,18-21,23-27,29,30]. Only one study reported bacterial isolation rates from cerebrospinal fluid cultures, with a 4% positivity rate [24]. Of all BI articles, two reported antibiotic use prior to blood culture, with 16% and 67% exposure rates, respectively [10,27] and two studies excluded those with prior antibiotic exposure [15,16].

### Laboratory methods and antibiotic susceptibility testing guidelines

Two of the 19 BI studies with blood cultures reported taking two blood samples from patients [14,15]. Of these 19 studies, 13 reported blood quantity taken [10-13,15-19,21,23,25,27]. Overall, ten of 12 studies retained in the AR analysis (see Figure 1) cited the use of established guidelines for resistance interpretation [11,12,19,20,24-26,28-30] with the majority referring to the Clinical and Laboratory Standards Institute (CSLSI) methods. Two studies cited external quality control measures [24,27] and only Darmstadt et al. [27] sent samples to a reference lab for confirmation.

### Bacterial infection incidence estimates and pathogens

**Figure 2:**
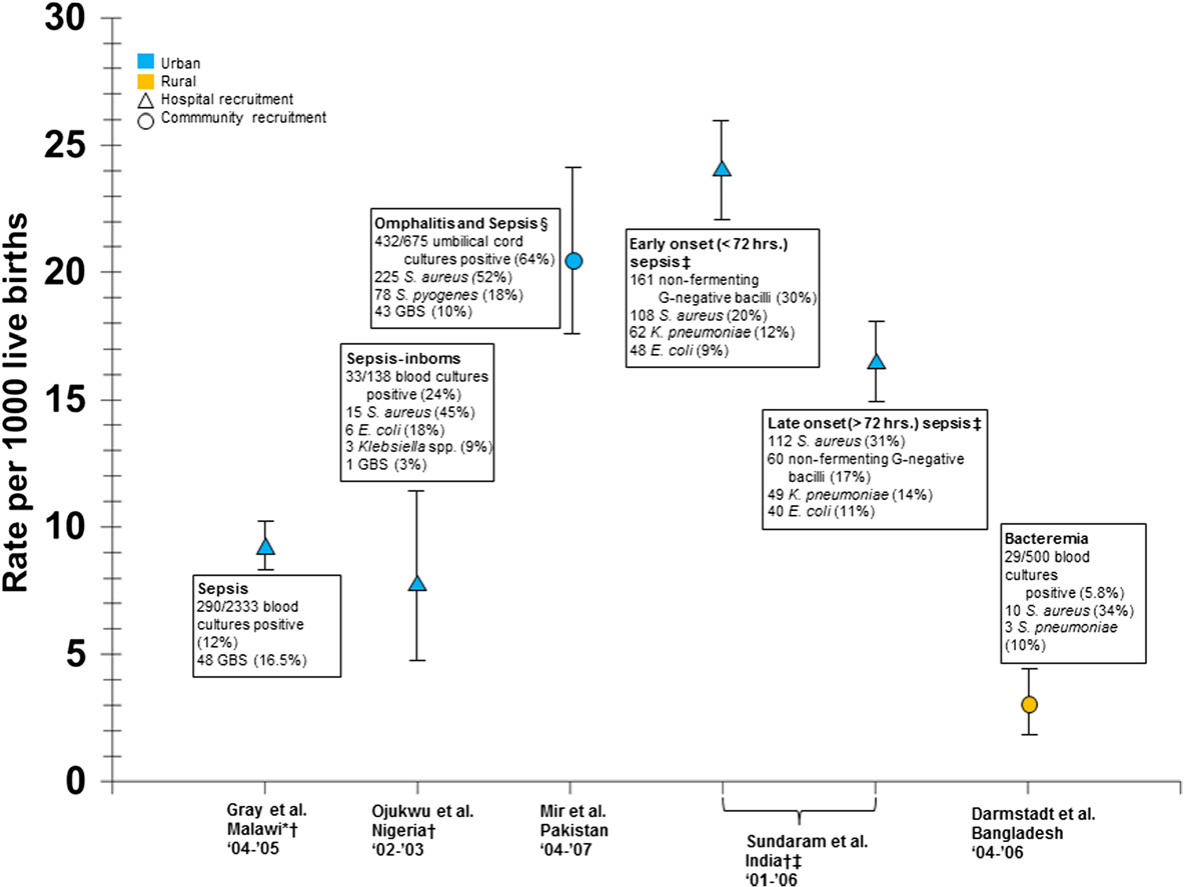
**Incidence and aetiology of neonatal sepsis/bacteremia for 1000 live births in developing countries.** *Sources;* [18,23,27-29] The figure shows point estimates, 95% confidence intervals, and aetiology of neonatal infections along with recruitment strategy and setting. Studies represented in blue were conducted in urban areas. Studies represented in orange were conducted in rural areas. Studies represented by a triangle used hospital recruitment. Studies represented by a circle used community recruitment. GBS (Group B streptococcus). *The Incidence estimate was calculated from the number of isolates and births presented. †CI estimated from data presented in the article. ‡The two estimates were taken from the same study. § Each case had both omphalitis and clinically defined sepsis.

Of 20 studies of BI in neonates, five reported an incidence rate for invasive bacterial infection (Figure 2) [18,23,27-29]. Incidence rates per 1000 live births ranged from 2.9 (95% CI 1.9–4.2) for bacteremia among neonates in Bangladesh [27] to 24 (95% CI 21.8–25.7) for early onset sepsis (<72 hours) in India [23]. Along with Darmstadt et al. [27], Mir et al. [28] used community recruitment and reported an incidence of 20.4 (95% CI 17.3–24.0) in Pakistan for neonatal omphalitis with sepsis [28]. Studies based on hospital recruitment reported sepsis incidence rates of 9.2 (95% CI 8.2–10.3) in Malawi [29] and 7.8 (95% CI 4.4–11.5) in Nigeria [18] per 1000 live births. A third study using hospital recruitment in India reported rates of 24 (95% CI 21.7–25.7) for early onset sepsis (<72 hours), and 16 (95% CI 14.0–18.1) for late onset sepsis (>72 hours) [23].

Among the 20 neonatal studies reporting full bacterial etiology, *Staphylococcus aureus* (*S. aureus)* was reported in all but two, accounting for 3% to 63% (median = 32.5%) of pathogens. Other common pathogens included *Klebsiella* spp*.*, reported in 16 studies and ranging from 8% to 66% (median = 22%) of pathogens, and *Escherichia coli (E. coli),* reported in 14 studies and ranging from 5% to 23% (median = 12%) of isolated pathogens. Sundaram et al. [23] reported that non-fermenting Gram-negative bacteria were the most common isolates in early-onset sepsis (first 72 hrs. of life) representing 30% of isolates, followed by *S. aureus* (20%), *Klebsiella pneumoniae* (*K. pneumoniae)* (12%), and *E. coli* (9%), whereas *S. aureus* predominated in late-onset sepsis (31%), followed by non-fermenting Gram-negative bacteria (17%), *K. pneumoniae* (14%), and *E. coli* (11%) [23]. Blomberg et al. [10] and Mhada et al. [11] reported results for early-onset sepsis defined as 0–7 days. Both studies found early onset sepsis was due primarily to *Klebsiella* spp. (33% and 32% respectively), *S. aureus* (29%, 11%) and *E. coli* (19%, 11%) with late-onset sepsis, defined as 7–28 days and 7–30 days respectively, due mostly to *S. aureus* (55%, 16%), *Klebsiella* spp. (23%, 23%), and *E. coli* (18%, 10%).

Twelve studies reported Group B *streptococcus* isolates. Percentages were low overall, representing between 1% and 20% (median = 4.5) of blood culture isolates in eleven studies [10,11,13-15,18,21,24,25,27-29].

### Antibiotic resistance

The 12 studies with relevant antibiotic resistance information for selected neonatal pathogens are presented in Table 3. Seven of the 12 studies were conducted before 2008. Resistance to penicillin/ampicillin among Gram-negative bacteria (not including *Klebsiella* spp.) ranged from 55% (95% CI 26%-84%) among *E. coli* isolates in Georgia [14] to 100% among *E. coli* isolates in Uganda [15]. Resistance to gentamicin among Gram-negative bacteria ranged from 0% for *Pseudomonas* and *E. coli* in Pakistan [28] and for *K. pneumoniae* in Nepal [20] to 100% for *K. pneumoniae* in India [25]. Among Gram-negative bacteria, resistance to third generation cephalosporins (3^rd^ GC) ranged from 6% for *E. coli* isolates in Uganda [15] to 97% among *K. pneumonia* isolates in India [25]. Only two studies tested for extended spectrum beta-lactamase (ESBL) production in *Enterobacteriaceae*. One reported ESBL production in 87% of *Klebsiella* spp. isolates, 73% of *Enterobacter* spp. isolates, and 65% of *E. coli* isolates [26]. The second found 32% ESBL production among *K. pneumoniae* isolates [25]. Resistance of *S. aureus* isolates to methicillin was reported in five studies and ranged from 0% to 67% [14,19,24,28,30].

**Table 3:** **Antibiotic resistance of bacteria isolated from invasive neonatal infections in developing countries (2000-May 2014)**

**Study, year**	**Location, setting**	**Pathogen**	**Resistant% (95% CI)**			
			**Penicillin/ampicillin**	**Gentamicin**	**3rd generation cephalosporins**	% ESBL*
Mugalu et al. [15]	Uganda, urban	17 *E. coli* ^†^ *,* ^‡^	100^§^	29 (7–51)	6 (0–17)	--
2002		7 Group B *Streptococcus* ^†^	14 (0–40)	57 (20–94)	--	NA
Gray et al. [29]	Malawi, urban	57 Group B S*treptococcus* ^†^	0^§^	--	0^§^	NA
2004-2005						
Shitaye et al. [19]	Ethiopia, urban	30 *S. aureus*	67% (50–84) resistance to meticillin			NA
2006-2007						
Talbert et al. [24]	Kenya, rural	48 *Acinetobacter* spp.^†^, ^||^	56 (42–70)	27 (14–39)	35 (22–48)	--
2001-2009		49 *K. pneumoniae* ^†^	96 (91–100)	49 (35–63)	43 (29–57)	--
		39 *S. pyogenes* ^†^	0^§^	--	--	--
		41 *E. coli* ^†^	78 (65–91)	10 (1–19)	17 (5–29)	--
		55 *S. aureus* ^†^	0% resistance to meticillin^§^			NA
Mhada et al.	Tanzania, urban	22 *Klebsiella* spp. ^||^	100^§^	77 (57–90)	18 (7–39)	--
2009-2010		41 *E. coli*	93 (69–99)	43 (21-67	14 (4–40)	--
Kruse et al. [30]	Vietnam, urban	78 *Klebsiella* spp. ^†^,^||^	100^§^	85 (75–91)	86 (76–92), 71 (60–79) ^¶^	--
2009-2010		58 *Acinetobacter* spp. ^†^,^||^	85 (73–92)	50 (38–62)	82 (71–80), 71 (58–81) ^¶^	--
		21 *E. coli* ^†^	86 (65–95)	57 (37–76)	58 (37–76), 42 (24–63) ^¶^	--
		16 *Enterobacter* spp. ^†^,^||^	93 (72–99)	62 (39–82)	62 (39–82), 50 (28–72) ^¶^	--
		6 *Pseudomonas* spp. ^†^,^||^	100^§^	48 (19–81)	83 (44–97) ,33 (10–70) ^¶^	--
		11 *S. Aureus* ^†^	55% (28–79) resistance to meticillin			
Jain et al. [26]	India, urban	86 *Klebsiella* spp. ^||^	100^§^	89 (82–96)	63 (53–73), 49 (38–60) ^¶^	87 (80–94)
2001-2002		80 *Enterobacter* spp. ^||^	100^§^	93 (87–99)	64 (53–75), 54 (43–65) ^¶^	73 (63–83)
		49 *E. coli*	96 (91–100)	90 (72–98)	65 (52–78), 41 (27–55) ^¶^	65 (52–78)
Zakariya et al. [25]	India, urban	33 *K. pneumoniae* ^†^	--	100^§^	97(85–99), 97(85–99) ^¶^	32 (20–50)
2004-2006						
Mir et al. [28]	Pakistan, urban	52 *Pseudomonas* spp. ^†^,^||^	--	0^§^	--	--
2004-2007		12 *Klebsiella* spp^†^,^||^	--	8 (0–23)	8 (0–23)	--
		9 *E. coli* ^†^	--	0^§^	11 (0–31)	--
		304 *S. aureus* ^†^	4% (2–6) resistance to meticillin			
Gyawali et al. [12]	Nepal, urban	82 Enterobacteriacea ?	94 (87–97)	70 (59–78)	83 (73–90), 79 (69–87), 87 (78–92) ^¶^	--
2009-2010		21 *Pseudomonas* spp. ^||^	--	37 (21–59)	47 (28–68), 71 (50–86), 67 (45–82) ^¶^	--
		30 *Acinetobacer* spp. ^||^	--	56 (39–73)	53 (36–70), 65 (46–78), 73 (56–86) ^¶^	--
Shresta et al. [20]	Nepal, urban	8 *K. pneumoniae*	38 (14–69)	0^§^	--	--
2011-2012						
Macharashvili et al. [14]	Georgia, urban	45 *Klebsiella* spp. ^†^,^‡^, ^||^	98 (94–100)	11 (2–20)	16 (5–27), 18 (7–29) ^¶^	--
2003-2004		11 *E. coli* ^†^,^‡^	55 (26–84)	18 (0–41)	9 (0–26), 9 (0–26) ^¶^	--
		15 *S. aureus*	40% (15–65) resistance to meticillin			

*Extended-spectrum beta-lactamase.

^†^Results were presented for sensitivity, resistance calculated as 100 minus% sensitive.

^‡^Penicillin results based on amoxicillin.

^§^Calculation of a CI was impossible.

^||^Pathogens marked spp. means no further characterization was presented.

^¶^Multiple 3GCs were tested.

## Discussion

Our results highlight the dramatic lack of data on bacterial resistance patterns in neonatal infections in developing countries. They also underscore the paucity of reliable and convincing data on the burden of community-acquired invasive bacterial infections in newborns in these countries. This was pointed out by Berkley et al. almost ten years ago and more recently by Lubell et al. in 2009, demonstrating how little progress has been made on this issue [31,32]. These gaps in knowledge impede the improvement of prevention and treatment strategies of neonatal infections in these settings where the risk of neonatal death is the highest.

We observed a broad range of neonatal infection incidence estimates. This heterogeneity has been previously noted by other authors [33,34] and emphasizes the need for routine surveillance across settings to accurately estimate the incidence and monitor trends of neonatal bacterial infections.

We found that the most common pathogens were *S. aureus, Klebsiella* spp., and *E. coli* which account for almost two thirds of neonatal sepsis cases. This proportion is in line with others reviews conducted in DCs [34,35]. Care must be taken when interpreting these results, however, as differentiation of infections into community-acquired or hospital-acquired during the neonatal period can be difficult in DCs [36]. As a result, a small number of nosocomial infections may be included in our findings, thus influencing the pathogen distribution. Five studies were included in our analysis which reported coagulase-negative Staphylococci as responsible for a significant proportion of neonatal infection, however positive blood cultures for this pathogen may commonly be due to sample contamination.

The relative importance of the most common pathogens differs according to disease onset (early vs. late), however this distinction was not detailed in the majority of the studies. Early onset infections are generally attributed to pathogens transmitted from the vaginal or rectal flora of the mother to the child, while late onset infections are attributed to bacteria acquired from the infant’s surroundings (hospitals or community) with *S. aureus* and *Klebsiella* species more frequently implicated in hospital infections [37]. Infection control measures designed to prevent the acquisition of bacteria from the environment do not affect pathogens that are acquired at birth. Therefore, distinguishing maternal from environmental infection sources would allow for improved implementation of preventive strategies in these settings.

Our results show that since 2000 few studies with reliable data focused on resistance patterns. In addition, the number of isolates per study was generally very small: three-quarters of the 12 studies reporting resistance rates reported results based on fewer than than 30 isolates. The WHO recommends ampicillin and gentamicin as first-line treatment of neonatal sepsis unless there is infection of the skin or umbilicus (possible *S. aureus*), when cloxacillin is substituted for ampicillin. Our review shows that resistance to ampicillin was high. Data on gentamicin were heterogeneous and no clear conclusion could be drawn. However, the findings of our review are consistent with others studies [35] and confirm a trend of growing resistance to this drug combination.

Data on resistance to third generation cephalosporins were heterogeneous except among *Klebsiella* spp. for which notable resistance rates were reported [25,26]. Moreover, only two studies reported testing for extended spectrum beta-lactamase (*ESBL*) despite the fact that ESBL have been reported worldwide. Medication required to treat ESBL-producing *Enterobacteriaceae* is expensive and unaffordable for the majority of the population in these settings making these bacteria difficult to treat. It is therefore of the utmost importance to make reliable data available to guide strategies devoted to limiting the spread of ESBL pathogens in DCs.

Importantly, no conclusions can be drawn regarding methicillin resistant *S. aureus* (MRSA), despite the fact that this pathogen may be the first cause of neonatal infection. Furthermore, only one of the six studies in our review describing MRSA infection was conducted in a community setting. Community-associated MRSA (CA-MRSA) has emerged in the developed world and represents a growing problem. Data on CA-MRSA are scarce in DCs, despite the existence of risk factors associated with drug resistance in the community, such as over-the-counter antibiotics use, overcrowding, and poor hygiene are highly prevalent [38].

Our review highlights the scarcity and heterogeneity of the available data on both the incidence of invasive bacterial infection and resistance patterns. While these findings may reflect true differences in the burden of neonatal infection and among resistance patterns, they may also be explained by major differences in data-collection methods. The currently available data are thus insufficient to draw a true picture of the burden of invasive bacterial infections and resistance. Furthermore, the reported infection rates are likely to underestimate the true incidence. Three-quarters of the studies reviewed took place in urban settings with recruitment at large or teaching hospitals. In DCs, the majority of families do not seek care in hospitals, particularly in rural areas, because of resource constraints, distances to their homes, or differences in health care seeking behaviours [39]. This is particularly true for early onset neonatal sepsis in the context of home deliveries. Along with underestimating the incidence, these factors undoubtedly play a role in the low detection rates of Group B *Streptococcus* in DCs as these infections generally occur in the few hours after delivery. These results are contrary to those from developed countries where Group B *Streptococcus* is the major cause of neonatal sepsis [40,41].

The observed heterogeneity among incidence rates may also be explained by the difficulty of estimating these rates. Such estimates require population surveillance systems, which are expensive and time-consuming and are often lacking in DCs. Indeed, less than one-fifth of the studies reviewed were based on active surveillance. An additional factor is the difficulty to confirm diagnosis with blood culture. A positive blood culture requires adequate blood volume drawn in strict aseptic conditions by skilled staff at the right moment along with access to appropriate laboratory equipment and techniques accessible almost exclusively in large or teaching hospitals in DCs [42,43]. Furthermore, only two studies performed two blood cultures despite an increased chance of pathogen isolation. The proportion of antibiotic usage prior to blood culture performed at the hospital was also high among studies reporting. Thus, a single negative blood culture cannot completely rule out an infection and a substantial proportion of non-microbiologically confirmed sepsis cases potentially represents false-negatives [44].

Assessment of antibiotic resistance was often based on few isolates collected over several years, which is clearly insufficient to describe trends in bacterial resistance to antibiotics. Almost one-third of the studies reviewed did not refer to any guidelines for interpretation of antibiotic susceptibility, which may call into question the reliability of their results. Given the heterogeneity in antimicrobial susceptibility references, comparisons between studies are difficult. Of note, almost half of the studies reviewed were conducted in Africa. The observed lack of studies in Southeast Asia is alarming as the population in this area is greater than in Africa.

The relative paucity of reports on antibiotic resistance collected after 2008 is of particular concern in a context of rapidly evolving resistance profiles and emerging antibiotic resistance mechanisms. Real-time data are required to provide an accurate understanding of drug sensitivity and resistance patterns [5]. Although a potential explanation may be that recent data collected is less likely to be published, the period of time that has elapsed since 2007 is largely sufficient to reveal a decline in publications.

A recent study published following our last search date deserves to be mentioned. This study included 8889 infants under 2 months brought to health facilities for illness in 6 DCs. Blood culture was performed for more than 10% of these children and antibiotic susceptibility testing was conducted on isolated bacteria. Pathogen distributions and antibiotic sensitivity patterns were similar to our findings [45].

In its first report on global antimicrobial resistance, with data from 114 countries, the WHO found that resistance to seven common bacteria has reached alarming levels in all regions of the world [6]. It also highlights that many gaps exist in documentation of pathogens of major public health importance. Our analysis is in line with the WHO’s conclusion on the need for methodological standards to investigate these issues. The WHO report also draws attention to the fact that resistance may be overestimated in the general population as most reported samples were collected in large hospitals, consistent with our observation that data from the community are lacking. Finally, the WHO calls for actions to strengthen and coordinate collaboration to address these knowledge gaps.

Effective surveillance systems or research programs devoted to anti-infective resistance in infectious diseases such as tuberculosis, malaria, or HIV have been implemented over the past few years with the active support of various stakeholders (donors agencies, governments, research institutes). These systems have been able to provide reliable data allowing for the promotion of global action. International alliances to contain antibiotic resistance in DCs exist and have called for several actions including global research and surveillance, public health advocacy, and consumer and practitioner education. However, current research projects are often based in large or teaching hospitals, and drug resistance patterns and trends in antibiotic use are based on data from these hospitals. It is therefore imperative to accurately assess the burden of antibiotic-resistant infections in DCs, particularly among children as they bear the highest burden.

## Conclusion

Despite the recent global awareness of bacterial resistance issues and indications of the growing antibiotic resistance in DCs, epidemiological evidence remains limited and available data are not sufficient to draw a true, recent, and accurate picture of antibiotic resistance in DCs among neonates and particularly in the community.

Neonatal bacterial diseases are a major cause of death in these countries, and the risk of bacterial resistance emergence and dissemination is exacerbated by poor antibiotic control and precarious living conditions. Without data to evaluate the burden of antibiotic resistance in this population, the public health problem will undoubtedly remain underserved. Future research should be able to collect quality, standardized epidemiological data along with a reliable bacteriological diagnosis at the community level in order to allow for adapted public health measures necessary to combat antibiotic resistance.
